# Empyema of the Antrum of Highmore

**Published:** 1891-04-11

**Authors:** 


### EMPYEMA OF THE ANTRUM OF HIGHMORE.

The methods of diagnosing pus in the antrum are very uncertain and unsatisfactory. Hitherto we have had to depend on such points as the protrusion of the maxillary wall, increase of the discharge when the head is tilted to the opposite side, pain below the orbit, and swelling of the cheek; and to confirm the diagnosis and exploratory aspiration of the cavity was necessary. As a rule, however, the presence of pus in the middle meatus, which pus on being wiped away is immediately replaced, is, according to Greville Macdonald (Med. Annual, 1891), strong presumptive evidence of there being pus in either the frontal or maxillary sinus, or that the discharge is flowing from the anterior ethmoidal cells. Ziem lays stress on the fact of the disease being unilateral, and that the suppuration is intermittent. Moreau Brown, of Chicago, advises an injection into the antrum of a solution of peroxide of hydrogen through the hiatus semilunaris, when, if pus be present, it is driven out and fills the nose as a white foam. Voltilini has recently advocated illumination by putting an electric lamp inside the patient's mouth, when, if the antrum contains pus a dark shadow is seen in the lower part of the antral region of the cheek, and the upper portion appears red up to the orbit. If it is a case of cyst with serous contents the light passes through it readily, and the whole antrum is illuminated. Heryng uses for this purpose Reineger's electric spatula with an Edison lamp of five volts, the lingual portion having a vulcanite spatula. Christopher Heath has pointed out the fact that the thickness of the bones on the two sides may vary, and this may constitute a difficulty in arriving at a positive diagnosis in employing this method. Greville Macdonald has also found that any obstruction in the lumen of the nasal passages materially affects the transmission of light through the antrum, and in one of his cases one side was so dark from the extra thickness of the bone on that side that he was induced to open the antrum, although there was no strong corroborative evidence; but the antrum contained no pus. Therefore, he states, that this new means of diagnosis must be considered of service only as adding a little weight to the evidence when the latter is almost though not quite conclusive.

